# Music and Dementia: Exploring Protective Factors for Cognitive Function

**DOI:** 10.1093/geroni/igab046.2766

**Published:** 2021-12-17

**Authors:** Sebastian Santana, Michael Willden, Debra Sheets, Andre Smith, Robert Stawski, Stuart MacDonald

**Affiliations:** 1 University of Victoria, Victoria, British Columbia, Canada; 2 Oregon State University, Corvallis, Oregon, United States

## Abstract

Ample research suggests that musical interventions have the potential to boost social connection, engender positive emotions, and potentially buffer against depression in people with dementia (PwD). Here, our focus concerns expanding the present body of knowledge by quantifying the benefits of a music-based nonpharmacological intervention. The Voices in Motion (ViM) choir is an intergenerational sociocognitive lifestyle intervention designed to support caregivers and PwD. Over the course of 18 months, the well-being of PwD and caregiver dyads (N = 32; mean age = 79.6 years; 53% female) were rigorously assessed using an intensive repeated measures design. This project set out to determine whether positive change in the social dimensions of health (i.e., social connection [SC] and psychological well-being [WB]) ameliorates depression in PwD. Multilevel modeling was employed to examine longitudinal change within and between individuals. SC significantly predicted intraindividual change (□20 = -0.48, p =.03), with a predictive trend for between person differences (□00 = -0.58, p =.08). On occasions when PwD reported more SC, relative to their own baseline, they also reported fewer depressive symptoms. The effect associated with WB was significant at the between-person level (□00 = -0.18, p =.01). Our analysis suggests that a lifestyle intervention targeting psychological health and wellbeing may also contribute to the depressive signs and symptoms in PwD. As the current health care system is forced to adapt to social distancing and constant precautionary measures, it is crucial to understand and potentially harness the protective effects of modifiable lifestyle factors.

